# Nicotinamide Riboside Enhances Endothelial Precursor Cell Function to Promote Refractory Wound Healing Through Mediating the Sirt1/AMPK Pathway

**DOI:** 10.3389/fphar.2021.671563

**Published:** 2021-05-12

**Authors:** Zhen-hua Wang, Xiao-gang Bao, Jun-jie Hu, Si-bo Shen, Guo-hua Xu, Ye-lin Wu

**Affiliations:** ^1^ Tongji University Cancer Center, Shanghai Tenth People's Hospital, Tongji University School of Medicine, Shanghai, China; ^2^ Department of Laboratory Medicine, Changzheng Hospital, Naval Medical University, Shanghai, China; ^3^ Department of Orthopedic Surgery, Spine Center, Changzheng Hospital, Naval Medical University, Shanghai, China; ^4^ Basic Medical College, Naval Medical University, Shanghai, China; ^5^ Hebei Key Laboratory of Active Components and Functions in Natural Products, College of Chemical Engi-neering, Hebei Normal University of Science and Technology, Qinhuangdao, China

**Keywords:** diabetes mellitus, endothelial precursor cells, nicotinamide riboside, wound healing, sirtuin 1, adenosine monophosphate–activated protein kinase

## Abstract

Lack of vascularization is directly associated with refractory wound healing in diabetes mellitus (DM). Enrichment of endothelial precursor cells (EPCs) is a promising but challenging approach for the treatment of diabetic wounds. Herein, we investigate the action of nicotinamide riboside (NR) on EPC function for improved healing of diabetic wounds. *Db/db* mice that were treated with NR-supplemented food (400 mg/kg/d) for 12 weeks exhibited higher wound healing rates and angiogenesis than untreated *db/db* mice. In agreement with this phenotype, NR supplementation significantly increased the number of blood EPCs and bone marrow (BM)-derived EPCs of *db/db* mice, as well as the tube formation and adhesion functions of BM-EPCs. Furthermore, NR-supplemented BM-EPCs showed higher expression of sirtuin 1 (Sirt1), phosphorylated adenosine monophosphate–activated protein kinase (p-AMPK), and lower expression of acetylated peroxisome proliferator–activated receptor γ coactivator (PGC-1α) than BM-EPCs isolated from untreated *db/db* mice. Knockdown of Sirt1 in BM-EPCs significantly abolished the tube formation and adhesion function of NR as well as the expression of p-AMPK and deacetylated PGC-1a. Inhibition of AMPK abolished the NR-regulated EPC function but had no effect on Sirt1 expression, demonstrating that NR enhances EPC function through the Sirt1-AMPK pathway. Overall, this study demonstrates that the oral uptake of NR enhances the EPC function to promote diabetic wound healing, indicating that NR supplementation might be a promising strategy to prevent the progression of diabetic complications.

## Introduction

The prevalence of diabetes mellitus (DM) and DM-related complications has been convincingly described regarding clinical, social, and economic implications (American Diabetes Association, 2018; Sørensen et al., 2016). By 2030, there will be approximately 430 million people with DM worldwide (Ginter and Simko, 2012). The incidence rate of diabetic foot ulcers (DFUs) in this cohort is increasing to around 20%, with an average annual cost of $8,659 for one patient in the United States (American Diabetes Association, 2018; Ginter and Simko, 2012). Diabetic patients with foot ulcers are at a high risk of operative limb salvage programs, including even major and minor amputations (Wukich et al., 2017). As lower extremity amputations are most often attributed to intractable DFUs, earlier prevention and treatment for patients with DFUs definitely play a critical role.

Delayed wound closure in DM patients is involved in peripheral circulatory disorders, which are closely related to neovascularization and angiogenesis (Forsythe and Hinchliffe, 2016; Hugo et al., 2016). It is well known that endothelial precursor cells (EPCs) play an important role in modified angiogenesis of the wound healing process (Han et al., 2017a). Since EPCs are rich in the peripheral blood of adults, there have been evidence-based demonstrations of migration to the damage site and formation of new vessels, as well as maintenance of vascular homeostasis (Catrina and Zheng, 2016). In recent years, EPC transplantation therapy has become a promising measure for ischemic diseases; remarkably, it could speed up the tissue repair process and ameliorate pathological conditions, including nutrient deficiency, inflammation, ischemia, and lower EPC numbers (Li et al., 2018; Zhao et al., 2018). Unfortunately, clinical evidence suggests that both EPC number and EPC function are impaired in patients with DM compared to healthy individuals (Wils et al., 2017). Therefore, several studies have shown that pharmacologically or molecularly modified EPCs are key strategies for enhancing their functionality and therapeutic effects (Ishida et al., 2012; Yu et al., 2016; Han et al., 2017a). In diabetic mice models, metformin and acarbose have been reported to promote wound closure by recruiting bone marrow (BM)-EPCs and improving their activity (Yu et al., 2016; Han et al., 2017b). Nevertheless, the mechanisms that underlie EPC-mediated wound healing have not been fully elucidated. Thus, it is imperative to investigate the mechanisms and novel agents in order to manage diabetic wound healing.

Nicotinamide riboside (NR), a new form of vitamin B3, acts as a nicotinamide adenine dinucleotide (NAD) precursor (Lee and Yang, 2019). NAD not only plays an essential role in cellular metabolism and energy production but also is a cofactor for sirtuin deacetylases (Lee and Yang, 2019). An extensive literature has indicated that supplementation of NAD can ameliorate age-related neurodegenerative diseases, nonalcoholic fatty liver disease (NAFLD), and metabolic disorders (Cantó et al., 2012; Zhou et al., 2016; Xie et al., 2019). Neiva et al. (2005) have reported that supplementation of vitamin B complex resulted in accelerated periodontal wound healing. In addition, Wang et al. (2016) have shown that depletion of the NAD pool is associated with the impairment of EPC mobilization in diabetes. NR has promising characteristics for Alzheimer’s disease, insulin sensitivity, and diabetic neuropathy (Cantó et al., 2012; Trammell et al., 2016; Xie et al., 2019). In addition, an oral supplement of NR is available, with the brand name NIAGEN®. However, whether replenishing NAD by supplementation of NR can enhance EPC function and improve wound repair remains unknown.

In recent years, accumulated evidence has demonstrated that dysregulation of adenosine monophosphate–activated protein kinase (AMPK)-mediated EPC dysfunction is associated with diabetic complications (Yu et al., 2016; Han et al., 2017a). Activated AMPK by metformin can enhance EPC numbers in circulation and improve the tube formation of EPCs. Moreover, inactivating the sirtuin 1 (Sirt1)/AMPK pathway has been observed in obese mice, which aggravates metabolic disorders (Francini et al., 2019). In obese mice, resveratrol as a Sirt1 activator could upregulate the expression of Sirt1 and phosphorylated AMPK (p-AMPK), inducing glucose metabolism (Tiao et al., 2018). Silence of Sirt1 could abolish the promoting effects of NAD on EPC proliferation and migration (Wang et al., 2014).

Therefore, we hypothesized that NR treatment may improve the EPC function to promote wound healing in diabetic conditions. In this study, we show that NR supplementation promoted angiogenesis and wound healing in *db/db* mice, and that NR regulated the EPC function to promote angiogenesis through mediating the Sirt1/AMPK pathway.

## Materials and Methods

### Animals

Four-week-old male C57BLKS/J *db/db* mice and age-matched C57BL/6J mice were purchased from the Laboratory Animal Center of Hangzhou Medical College (Hangzhou, China). All mice were housed in cages at 23 ± 2°C in a humidity-controlled holding room with a 12-h light/dark schedule. Mice were provided with free access to water and food. All mice were handled in accordance with the National Institutes of Health’s Guide for the Care and Use of Laboratory Animals.

### Experimental Protocols

**FIGURE 1:**
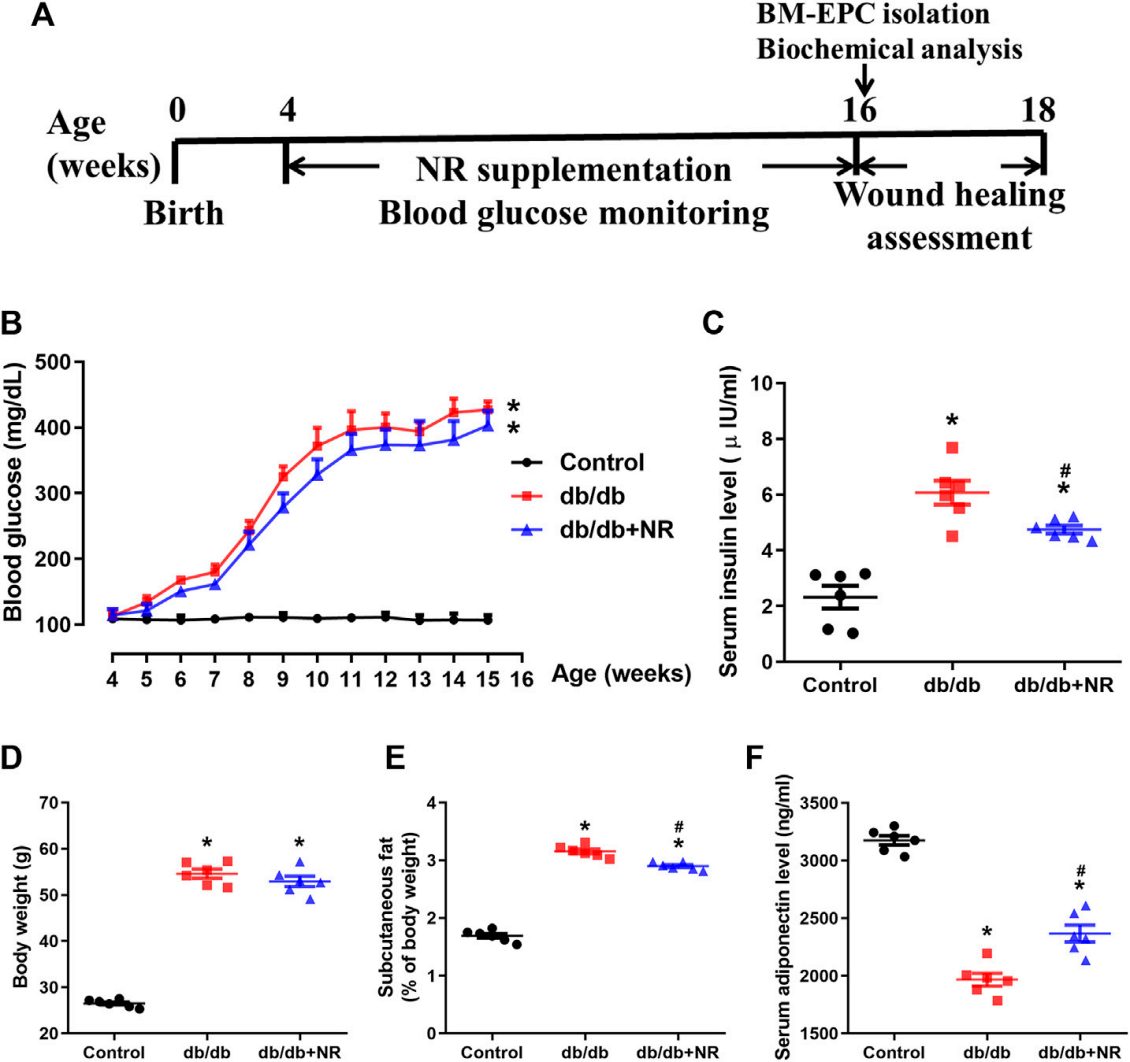
Effects of NR supplement on some diabetes mellitus–related symptoms. **(A)** Experimental schedule. *Db/db* mice (4 weeks old) were fed with common food or NR-supplied food (400 mg/kg/d) for consecutive 12 weeks, and the blood glucose was monitored every week. After that, each group mice were further divided into 2 cohorts: excisional wound experiment and BM-EPC isolation experiment. Blood glucose **(B)**, serum insulin **(C)**, body weight **(D)**, subcutaneous fat **(E)**, and serum adiponectin **(F)** of the mice treated with common food or NR-supplied food for 12 weeks. Values are mean ± SEM, (*n* = 6). *P < 0.05 *vs.* control; ^#^ *P* < 0.05 *vs. db/db*. BM-EPC, bone marrow–derived endothelial precursor cell.

The *db/db* mice were randomly separated into 2 experimental groups, either receiving common food or NR-supplemented food (approximately 400 mg/kg/day; Xie et al., 2019) for 12 weeks. NR was bought from Baikai Chemical Technology Co., Ltd. (Hangzhou, China, CAS: 1341-23-7) and mixed into the common food. The C57BL/6J mice received common pellets as a control group. Blood glucose was detected every week using a monitoring system (Maochang, Taipei, China). Whole blood samples of the mice were obtained from the tail veins. After 12 weeks of NR administration, body weight changes were determined in each group. Six mice of each group were used to produce a wound model and isolating of BM-EPCs (Figure 1A). At the end of the experiment, all mice were sacrificed by cervical dislocation.

### Sample Collection

Blood was collected by excising the eyeballs. About 0.6 ml of blood was harvested. One half was used for EPC isolation, and the remaining half was placed at room temperature (RT) for 40 min and at 4°C for 2 h. The samples were then centrifuged at 3,000 ×g for 10 min, and the supernatant was collected and stored at −80°C for index measurement. After blood collection, the subcutaneous fat was carefully dissected and weighed.

### Serum Insulin and Adiponectin Measurements

Serum insulin was determined using an ELISA kit from Shibayagi Co., Ltd. (Shibukawa, Japan). Serum concentration of adiponectin was also measured by commercial ELSA kits (adiponectin: AdipoGen, Incheon, Korea). All samples were measured according to the manufacturers’ protocols.

### Wound Closure Test

Mice were anesthetized with phenobarbital sodium (60 mg/kg, *i.p.*). A 6-mm circular wound was made by punch biopsy (Wu et al., 2016; Lei et al., 2017). The wounds were clipped off the full thickness and subcutaneous tissue. The surgery mice were individually caged and placed on heat mats until fully recovered. Digital images of the wound on the dorsum were captured every 2 days until the end of the experiment for all experimental mice. The wound areas were analyzed by tracing the wound margins and calculated using Image-Pro Plus software version 6.0 (Media Cybernetics, Rockville, MD, United States). The closure was expressed as a percentage area of the original wound area.

### Evaluation of Wound Angiogenesis

Mice were sacrificed by cervical dislocation after wound assay. The tissues around and under the wound were clipped off by scissors and incubated in 4% paraformaldehyde at RT overnight. Then, the tissues were transferred to 17% sucrose for 24 h at RT and embedded in paraffin for immunochemistry analyses to quantify the wound angiogenesis by CD31 staining (Han et al., 2017b). The fixed tissues were sliced into 5-μm-thick sections and blocked with 5% serum for 3 h. Samples were subsequently incubated with an anti-CD31 antibody (BD Biosciences, San Diego, CA, United States; 1:500, cat. no. 550274) for 1 h at RT and incubated with a secondary antibody (Vector Laboratories Ltd, Peterborough, UK; 1:800, cat. no. BA-9200) for 1 h each. The slides were counterstained with hematoxylin for another 2 min and were then examined under low-power fields (magnification ×100) and high-power fields (magnification ×200), respectively, by a light microscope (Leica, Wetzlar, Germany). A capillary was defined as CD31 positive staining, and the capillary numbers were calculated.

### Blood EPCs and BM-EPC Assessment

Blood EPCs and BM-EPC frequencies were assayed by flow cytometry, as described in a previous study (Yu et al., 2016). The peripheral blood or BM was isolated by Ficoll density gradient centrifugation. After the cell layer was extracted and red blood cells were lysed, the cells were stained with FITC-Sca-1 (BD, San Diego, CA, United States; cat. no. 557405) and PE-Flk-1 (BD, San Diego, CA, United States; cat. no. 555308) antibodies to measure the frequency of EPCs (Sca-1^+^/Flk-1^+^).

### BM-EPC Extraction

BM-EPC isolation was performed as previously described (Han et al., 2017a). Briefly, BM-EPCs were isolated from the femurs and tibias of mice from each group. Cells were maintained in endothelial growth medium-2 (EGM-2; Cambrex Corp, East Rutherford, NJ, United States) with 15% fetal bovine serum and incubated at 37°C with 5% carbon dioxide (CO_2_). The EGM-2 medium was replaced after 3 days, and cells were allowed to culture for another 4 days. BM-EPCs from different mice were pooled and then separated to a 12-well plate to evaluate the EPC function. In other cellular experiments, mature BM-EPCs were treated with the AMPK inhibitor compound C (10 μM; Sigma, St. Louis, MO, United States) or C75 (40 μg/ml; Sigma). After 24 h of compound C or 2 h of C75 stimulation, cell analysis was performed to evaluate the EPC functions. The doses and incubation time of compound C and C75 were referred to those in previous reports (Kim et al., 2004; Yu et al., 2016).

### Measurement of NAD and Vascular Endothelial Growth Factor Concentration

Measurement of NAD concentration was performed according to the method previously described (Wang et al., 2011). The level of NAD^+^ in the cellular experiment was assayed using a NAD Quantification Kit (BioVision, Mountain View, CA, United States). The level of VEGF in EPCs was also measured with a commercial ELSA kit (R&D System, Minneapolis, MN, United States). All samples were measured according to the manufacturers’ protocols.

### Determination of BM-EPC Function

A tube formation assay was performed to evaluate the BM-EPC function as described before (Yu et al., 2016). Briefly, a 96-well plate (Corning, Tewksbury, MA, United States) was coated with 50 μL/well of growth factor–reduced matrix gel (BD Biosciences, San Diego, CA, United States; cat. no. 356231) for 1 h. The cells were then placed on the Matrigel-coated plate with 5 × 10^5^/ml concentration and maintained at 37°C with 5% CO_2_. After 8 h of incubation, images of the forming tubes were acquired under ×50 magnification using a light microscope. The number of tubes was calculated.

The adhesion ability assay was used to assess the EPC function as previously described (Han et al., 2017a). A total of 5 × 10^4^ BM-EPCs were placed on the mouse vitronectin (1 μg/ml) coated 96-well plate per well. After 2 h incubation at 37°C with 5% CO_2_, nonadherent cells were softly removed by phosphate-buffered saline (PBS). Then, adherent cells were fixed with 2% paraformaldehyde for 15 min at RT and stained by Hoechst 33258 (10 μg/ml; Beyotime, Shanghai, China; cat. no. C1011). The stained cells (blue color) were observed using a fluorescence microscope (Leica, Wetzlar, Germany) at a magnification of ×400. Each well was counted in 3 random fields.

### Small Interfering RNA Transfection

Transfection of siRNA using Lipofectamine 2000 (Invitrogen, Carlsbad, CA, United States) was done as previously described (Wang et al., 2014). The commercial siRNA targeting Sirt1 was purchased from Dharmacon (Lafayette, CO, United States). All steps were strictly followed in accordance with the manufacturer’s instructions. After transfection for 6 h, the medium containing siRNA was replaced. The medium was cultured for another 2 days, and the cells were used for EPC function assays or Western blot analysis.

### Western Blot and Immunoprecipitation

For the Western blot assay, the samples were separated by 8% or 10% SDS–PAGE and blotted onto the polyvinylidene difluoride membrane by electrophoretic transfer (Bio-Rad Laboratories, Inc, Hercules, CA, United States). The members were probed with specific antibodies overnight at 4°C and then incubated with secondary antibodies at RT for 2 h. A quantitative analysis of the visualized bands was performed with Scion Image analysis software (Bio-Rad Laboratories). The specific primary antibodies were Sirt1 (Cell Signaling Technology, Danvers, MA, United States; cat. no. 8469S), phosphorylated AMPKα (p-AMPKα; Cell Signaling Technology; cat. no. 50081S), AMPKα (Cell Signaling Technology; cat. no. 5832S), and GAPDH (Sigma; cat. no. G9295).

For the immunoprecipitation assay, cells were lysed with RAPI buffer by adding protease inhibitors (Beyotime, Shanghai, China). Forty microliters of rabbit serum were added to 1 ml of lysates to decrease the nonspecific binding. The protein samples were incubated with protein G agarose for 2 h and centrifuged at 15,000 g for 10 min to obtain supernatant. This supernatant was incubated with peroxisome proliferator–activated receptor γ coactivator (PGC-1α; Sigma; cat. no. ABE868) primary antibody overnight at 4°C. The protein G agarose was used to harvest the protein complex. The immunoprecipitated protein was then blotted on 10% SDS–PAGE for immunoblotting. Acetylated PGC-1a was measured using acetyl-lysine antibody (Abcam, Cambridge, MA, United States; cat. no. ab190479).

### Statistical Analyses

**FIGURE 2:**
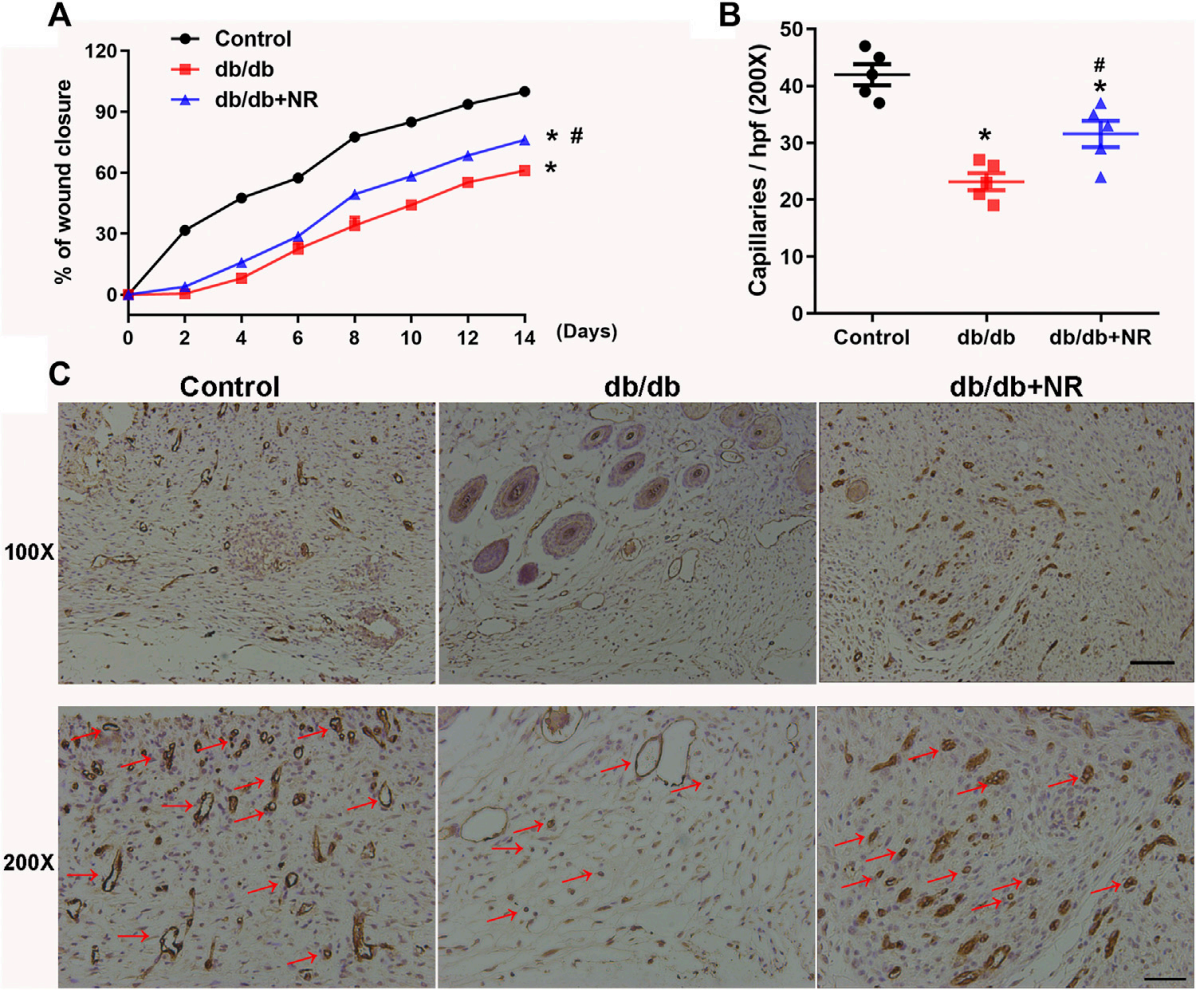
Effects of NR supplement on wound closure and wound angiogenesis in *db/db* mice. **(A)** Full-thickness skin wounds were made in common food and NR-supplied food fed *db/db* mice. Quantitative analysis of wound closure at indicated time intervals **(B)**. Immunohistochemical analyses of CD31 in day-14 wounds of mice and quantitative study **(C)**. Red arrows point out CD31-positive capillaries (100×, scale bar, 100 μm; 200×, scale bar, 50 μm). Values are mean ± SEM (*n* = 5). *P < 0.05 *vs.* control; ^#^ *P* < 0.05 *vs. db/db*.

Data were expressed as means ± SEM. Comparisons among the three groups were analyzed by one-way analysis of variance (ANOVA) followed by Tukey’s test using GraphPad Prism software (version 6). Comparisons between the two groups were performed using the unpaired Student’s *t*-test. Data in Figure 2A were analyzed by two-way ANOVA. Statistical significance was set at *P* less than 0.05.

## Results

### Effect of NR on Blood Glucose, Serum Insulin, Body Weight, and Serum Adiponectin Level in *db/db* Mice

To investigate the function of NR on wound healing in diabetic mice, the 4-week-old *db/db* mice were fed with NR-supplemented food for 12 weeks. As shown in Figure 1B, blood glucose was greatly increased in *db/db* mice compared with the age-matched normal C57BL/6J mice (*P* < 0.05). However, the NR supplement had little effect on blood glucose in *db/db* mice (*P* > 0.05; Figure 1B). Interestingly, the increased serum insulin in *db/db* mice was greatly reduced after NR treatment (4.75 ± 0.14 μIU/ml *vs.* 6.07 ± 0.43 μIU/ml, *P* < 0.05; Figure 1C). We also measured the body weight, subcutaneous fat, and adiponectin of the mice treated with or without NR. As shown in Figures 1D–F, the NR supplement had no significant effect on the body weight of *db/db* mice, but significantly decreased the subcutaneous fat ratio (2.90 ± 0.07% *vs.* 3.16 ± 0.10%, *P* < 0.05; Figure 1E) and increased adiponectin (2367 ± 73.31 ng/ml *vs.* 1967 ± 56.06 ng/ml, *P* < 0.05; Figure 1F) of *db/db* mice. These results suggest that NR supplementation has a minor effect on DM-related symptoms.

### NR Accelerated Wound Closure and Angiogenesis in *db/db* Mice

To investigate the role of NR supplementation in the wound healing process in diabetic mice, circular and full-thickness cutaneous wounds were created on the dorsal skin of mice, and the wound areas were measured every 2 days. As shown in Figure 2A, the healing rate of *db/db* mice was dramatically slower than that of normal mice; interestingly, NR supplementation significantly accelerated the healing rate of *db/db* mice (*P* < 0.05; Figure 2A).

Since the wound healing rate is highly correlated with the extent of angiogenesis, we assessed the vasculature of the wound edges using CD31 staining. As shown in Figures 2B,C, the number of CD31-positive vascular structures in the wounds of *db/db* mice was markedly less than that of normal mice (23.20 ± 1.50 *vs.* 42.00 ± 1.84, *P* < 0.05). However, NR treatment significantly increased the CD31-positive vascular structures (31.60 ± 2.32 *vs.* 23.20 ± 1.50, *P* < 0.05). These results suggest that NR supplementation might increase angiogenesis to promote wound healing in *db/db* diabetic mice.

### NR Increased EPC Number and Improved BM-EPC Function in *db/db* Mice

**FIGURE 3:**
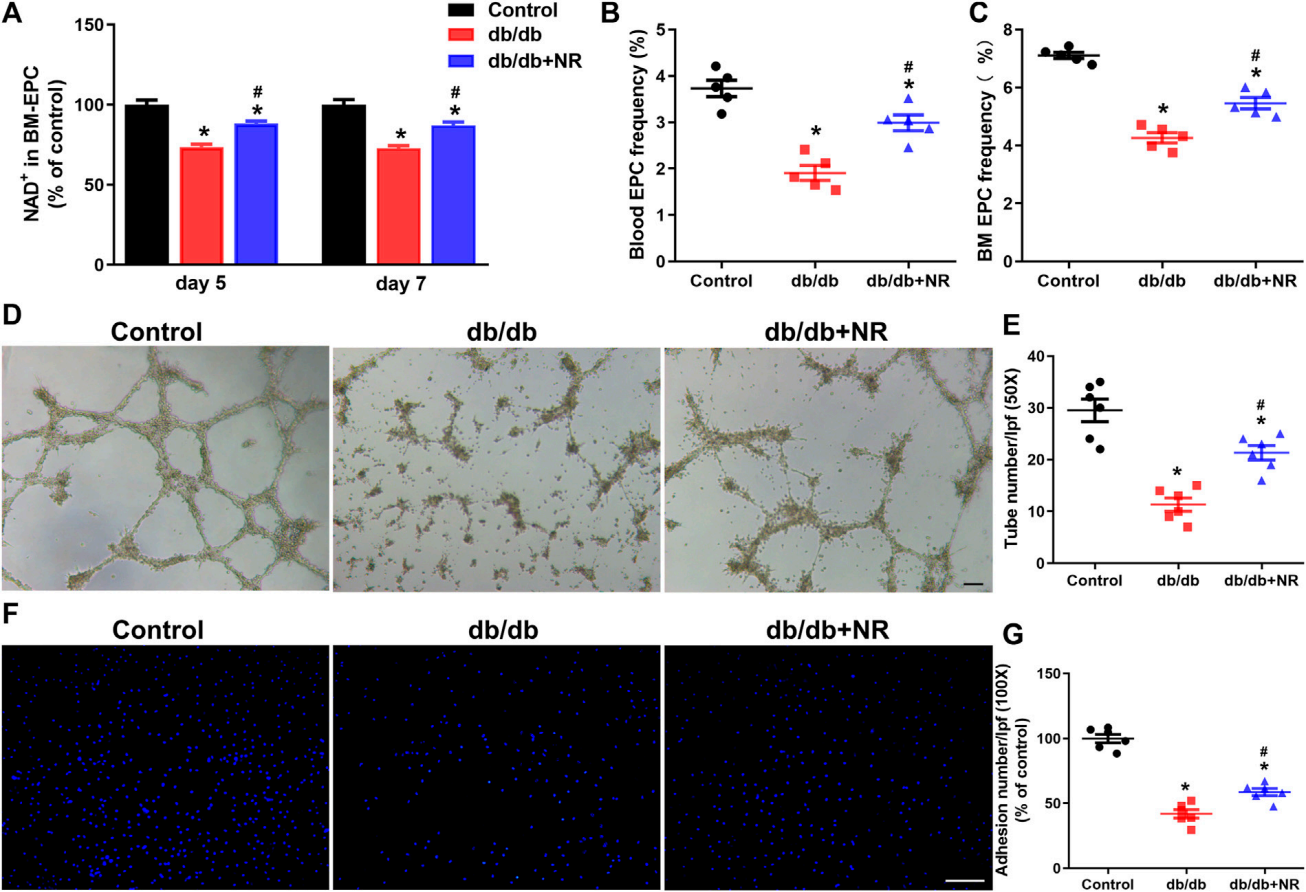
Effects of NR supplement on EPC number and BM-EPC function in *db/db* mice. **(A)** NAD concentrations in cultured BM-EPCs (day 5 and day 7 *in vitro*) isolated from mice. Blood EPC **(B)** and BM EPC **(C)** frequency in normal control and *db/db* mice. Tube formation **(D,E)** and cell adhesion **(F,G)** of BM EPC isolated from normal control and *db/db* mice. D: 50×; scale bar, 100μm; F: 100×; scale bar, 100 μm. Values are mean ± SEM (A, B, C: *n* = 5; D, E, F, G: *n* = 6). *P < 0.05 *vs.* Control; ^#^ *P* < 0.05 *vs. db/db*. BM, bone marrow; BM-EPC, bone marrow–derived endothelial precursor cell.

Previous studies have indicated that impaired angiogenesis in diabetic wounds is closely associated with the EPC number and function (Yu et al., 2016; Han et al., 2017a). Therefore, we isolated BM-EPCs and blood EPCs from *db/db* mice which were given common food and NR-supplemented food and then detected the NAD concentration, EPC number, tube formation, and cell adhesion function of BM-EPC *in vitro*. The concentration of NAD in cultured BM-EPCs (day 5 and day 7 *in vitro*) from *db/db* mice were remarkably lower than that of NAD in C57BL/6J mice (day 5: 73.50 ± 1.79% *vs.* 100.00 ± 3.00%, *P* < 0.05; day 7: 72.64 ± 1.77% *vs.* 100.00 ± 3.13%, *P* < 0.05; Figure 3A). However, NR successfully reversed the reduction of NAD concentration in BM-EPCs from *db/db* mice (day 5: 88.34 ± 1.42% *vs.* 73.50 ± 1.79%, *P* < 0.05; day 7: 87.06 ± 2.10% *vs.* 72.64 ± 1.77%, *P* < 0.05; Figure 3A). The EPC number in blood and in BM of *db/db* mice was reduced by 48% and 40%, respectively, compared with the control mice. NR supplementation greatly raised the frequencies of EPCs in blood (*P* < 0.05; Figure 3B) and BM EPCs (*P* < 0.05; Figure 3C) of *db/db* mice. Furthermore, tube formation (11.33 ± 1.28 *vs.* 29.50 ± 2.19, *P* < 0.05; Figures 3D,E) and cell adhesion function (41.97 ± 3.25% *vs.* 99.97 ± 3.28%, *P* < 0.05; Figures 3F,G) of BM-EPCs from *db/db* mice were significantly reduced compared with those of BM-EPCs from the control mice. NR supplementation also significantly elevated the tube formation (21.33 ± 1.38 *vs.* 11.33 ± 1.28, *P* < 0.05; Figures 3D,E) and adhesion ability (58.68 ± 2.73 *vs.* 41.97 ± 3.25, *P* < 0.05; Figures 3F,G) of BM-EPCs from *db/db* mice. These results suggest that NR supplementation might improve the BM-EPC function to promote angiogenesis, leading to accelerated wound healing.

### NR Enhanced SIRT1/AMPK Expression in BM-EPCs From *db/db* Mice

**FIGURE 4:**
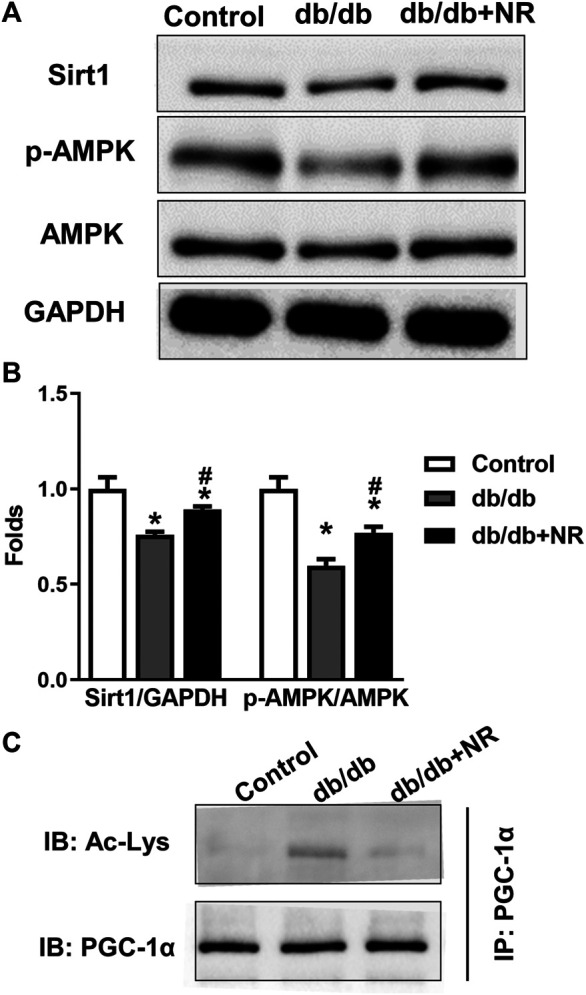
Effect of NR supplement on the expression of Sirt1/AMPK in BM-EPCs. **(A)** Western blot of Sirt1/AMPK in BM-EPCs isolated from normal control and diabetic mice. **(B)** Quantitative analysis of Sirt1 and p-AMPK expression taken as in **(A)**. **(C)** The level of acetylated PGC-1α was measured. Values are mean ± SEM, (*n* = 3). *P < 0.05 *vs.* Control; ^#^ *P* < 0.05 *vs. db/db*.

To confirm the potential mechanisms responsible for the therapeutic effect of NR supplementation on wound healing and angiogenesis, we detected the levels of Sirt1/AMPK, acetylated PGC-1α, and VEGF in BM-EPCs. As shown in the results of Western blot analysis, the downregulated Sirt1 and p-AMPK/AMPK expressions can be observed in BM-EPCs *from db/db* mice compared with those from the control mice (*P* < 0.05; Figures 4A,B). However, NR treatment significantly upregulated Sirt1 and p-AMPK/AMPK expressions in BM-EPCs compared with untreated cells from *db/db* mice (*P* < 0.05; Figures 4A,B). As PGC-1α deacetylation represents the activity of Sirt1, we performed immunoprecipitation WB experiment to detect acetylated PGC-1α. As shown in Figure 4C, the level of acetylated PGC-1α was obviously enhanced in BM-EPCs from *db/db* mice, which was inhibited after NR treatment, demonstrating NR enhances Sirt1 expression to deacetylate PGC-1α. Next, the level of VEGF in BM-EPCs was further evaluated. The results showed that VEGF concentration was significantly reduced in BM-EPCs *from db/db* mice compared with those from the control mice (415.9 ± 9.7 pg/ml *vs.* 638.3 ± 20.7 pg/ml, *P* < 0.05; Supplementary Figure S1A). Moreover, NR also increased the level of VEGF when compared to the cells from *db/db* mice (471.5 ± 15.4 pg/ml *vs.* 415.9 ± 9.7 pg/ml, *P* < 0.05; Supplementary Figure S1A). These results suggest that NR supplementation might promote BM-EPC function through increasing expression of Sirt1, deacetylated PGC-1α, p-AMPK/AMPK, and VEGF.

### Inhibition of Sirt1 Abolished the Protective Effects of NR Repletion on BM-EPC Function in *db/db* Mice

**FIGURE 5:**
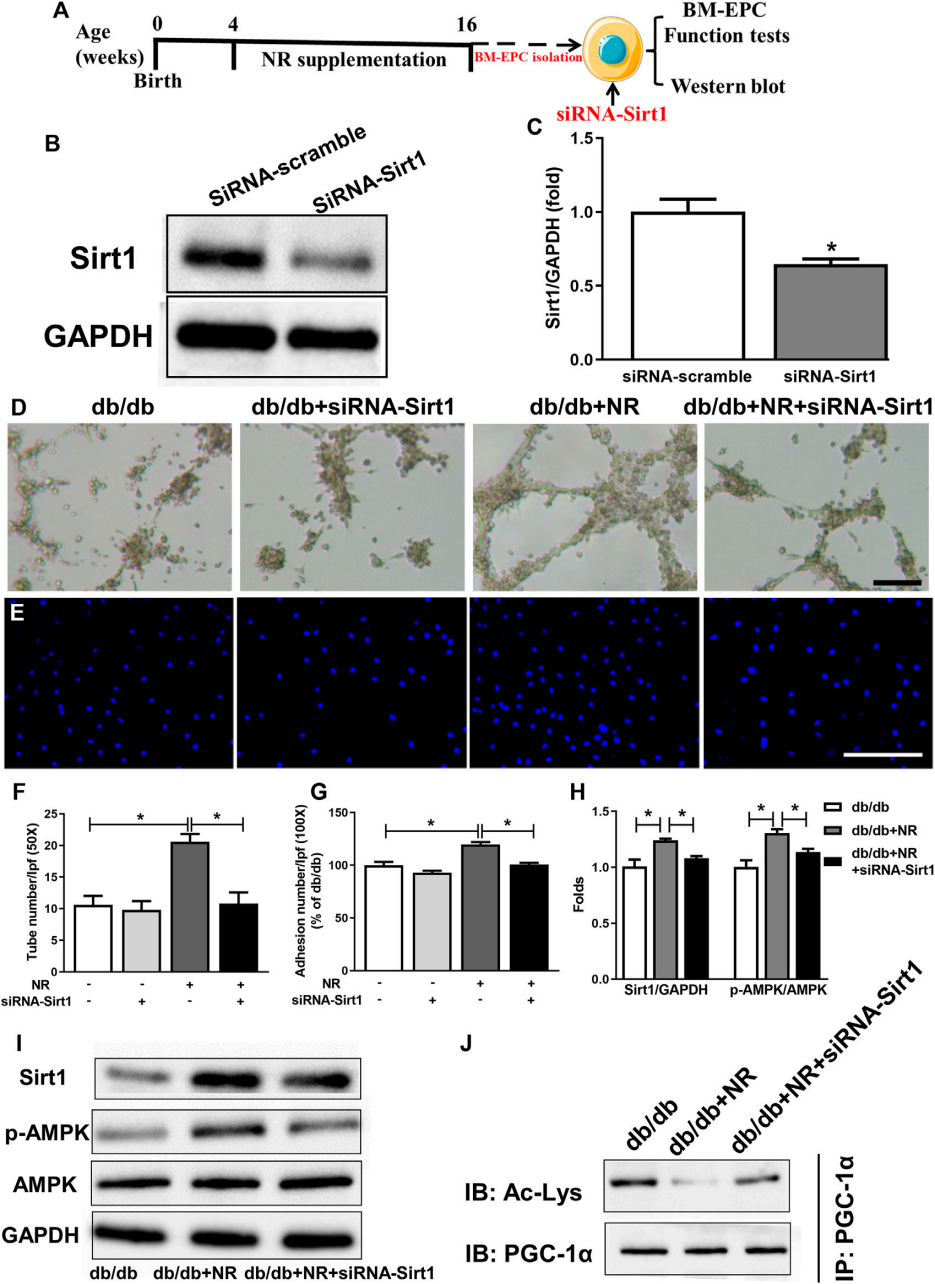
Inhibition of Sirt1 with siRNA-Sirt1 abolished the protective effects of NR repletion on BM-EPC function in *db/db* mice. **(A)** Experimental schedule. *Db/db* mice received the NR supplied food for 12 weeks, and then, BM-EPCs were isolated and transfected with siRNA targeting Sirt1. **(B,C)** Transfection of siRNA–Sirt1 obviously inhibited Sirt1 expression in BM-EPCs from *db/db* mice. *P < 0.05 *vs.* siRNA-Sirt1. Tube formation **(D)** and adhesion abilities of BM-EPCs **(E)** were measured. Quantitative analysis of tube number **(F)** and adhesion number **(G)**. **(H)** Quantitative analysis of Sirt1, and p-AMPK expression was taken as in **(I)**. **(I)** Western blot of Sirt1/AMPK in BM-EPCs treated with NR in the presence or absence of siRNA–Sirt1. **(J)** Representative immunoblot images of acetylated PGC-1α. D: 50×; scale bar, 100 μm; E: 100×; scale bar, 100 μm. Values are mean ± SEM, (B, C, H, I, J: *n* = 3; D–G: *n* = 5). *P < 0.05. BM-EPC, bone marrow–derived endothelial precursor cell; NS, no significance.

We next investigated how NR regulates Sirt1-mediated EPC function in diabetic mice. SiRNA was used to silence Sirt1 in BM-EPCs (Figure 5A). Sirt1 expression was significantly decreased using siRNA-mediated RNA interference in BM-EPCs (Figures 5B,C). Silencing of Sirt1 abolished the NR-enhanced tube formation (*P* < 0.05; Figures 5D,F) and adhesion abilities (*P* < 0.05; Figures 5E,G) in diabetic EPCs. Consistently, siRNA–Sirt1 treatment effectively inhibited NR-upregulated p-AMPK/AMPK and increased NR-inhibited acetylated PGC-1α (*P* < 0.05; Figures 5H–J). However, silencing of Sirt1 did not affect NR-increased VEGF in BM-EPCs of diabetic mice (*P* > 0.05; Supplementary Figure S1B). These results suggest that NR supplementation promotes Sirt1 expression to increase p-AMPK/AMPK and decrease acetylated PGC-1α expressions in BM-EPCs.

### Inhibition of AMPK Prevented the Effects of NR on BM-EPC Function in *db/db* Mice

**FIGURE 6:**
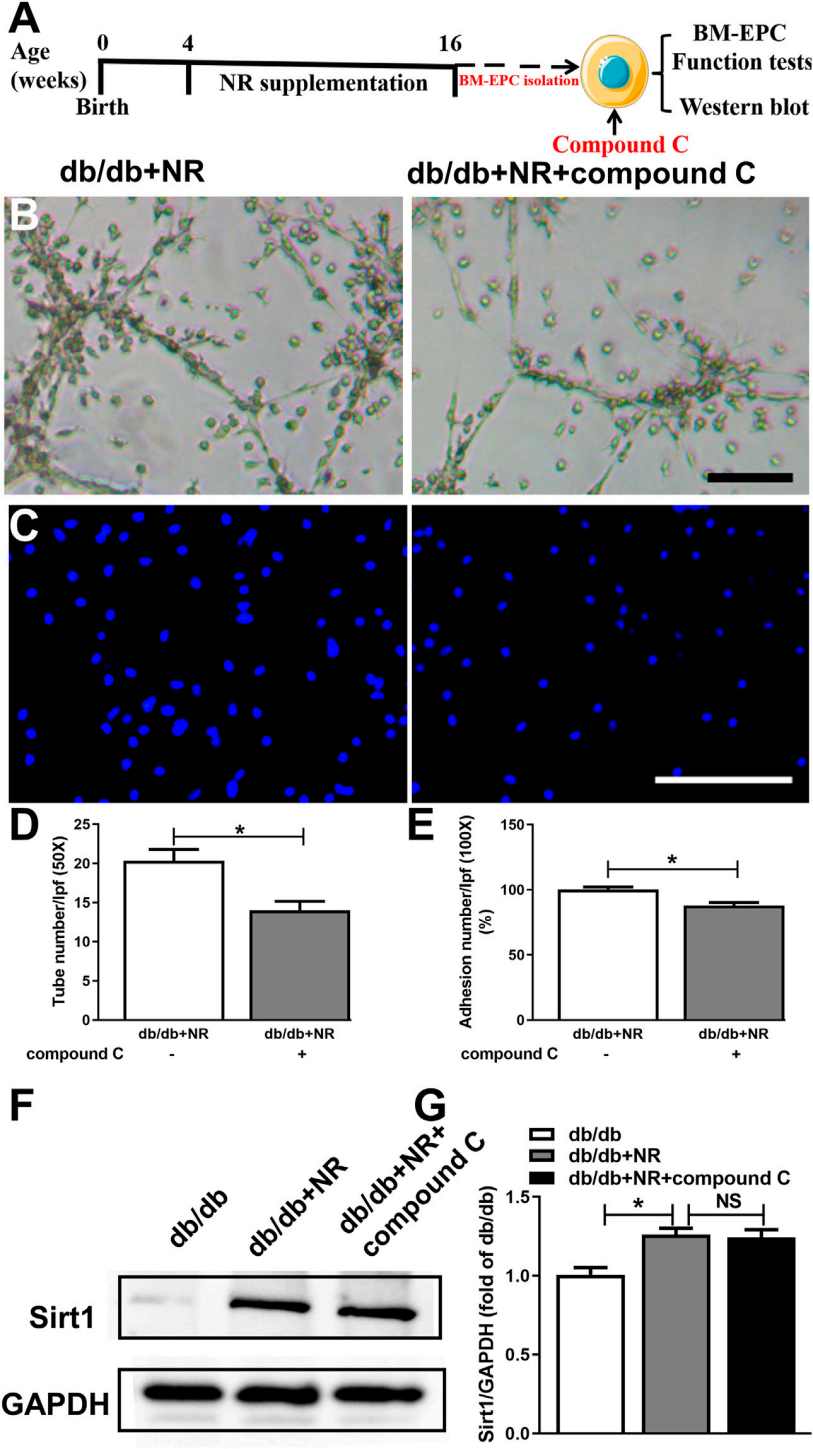
Inhibition of AMPK with compound C abolished the protective effects of NR supplement on BM-EPC function in *db/db* mice. **(A)** Illustration of experimental protocol. *Db/db* mice treated with NR for 12 weeks, and then, BM-EPCs were isolated and stimulated with compound C (10 μM). Tube formation **(B)** and adhesion **(C)** assay in BM-EPCs treated with NR in the presence or absence of compound C. Quantitated analysis of tube number **(D)** and adhesion number **(E)**. **(F–G)** Compound C has no significant effect on Sirt1 expression in BM-EPCs of *db/db* mice treated with NR. B: 50×; scale bar, 100 μm; C: 100×; scale bar, 100 μm. Values are mean ± SEM (*n* = 3). *P < 0.05. BM-EPC, bone marrow–derived endothelial precursor cell; NS, no significance.

To further investigate whether AMPK signal is needed for NR-regulated BM-EPC function, two important antagonists of AMPK (compound C and C75, Jiang et al., 2018) were used to analyze the NR-mediated BM-EPC function. Inhibition of AMPK by compound C or C75 significantly inhibited the NR-increased tube formation (*P* < 0.05; Figures 6B,D; Supplementary Figures S2B,D) and adhesion (*P* < 0.05; Figures 6C,E; Supplementary Figures S2C,E). These results suggest that AMPK signal is needed for the NR-regulated BM-EPC function. Furthermore, inhibition of AMPK by compound C had no significant effects on NR-induced Sirt1 expression (*P* > 0.05; Figures 6B–D), demonstrating that AMPK is downstream of the Sirt1 signaling pathway. Taken together, all these data suggest that NR enhances EPC function through mediating the Sirt1/AMPK pathway.

## Discussion

In the present study, we have shown that 1) *db/db* mice administered with NR exhibited accelerated wound healing and alleviated angiogenesis accompanied by an increased concentration of NAD^+^, 2) these improvements are closely associated with enhanced an EPC number and function in *db/db* mice, and 3) the beneficial effects of NR were mediated in part through the activation of Sirt1/AMPK. Taken together, we show novel evidence for the therapeutic effect of NR on diabetic wound healing by improvement of EPC function *via* activating the Sirt1/AMPK signaling pathway in *db/db* mice.

Previous reports indicated that *db/db* mice with a C57BLKS/J background, a mutation of the leptin receptor gene, widely served as a mouse model for type 2 DM (T2DM) (Zhong et al., 2013). To study the effect of NR on diabetic wounds, 4-week-old *db/d*b mice were used because they could rapidly develop hyperglycemia, dyslipidemia, and insulin resistance from 6 weeks of age, and DM-related complications commonly occur after 10 weeks of age (Zhong et al., 2013; Kang et al., 2014). In this study, we examined blood glucose and body mass in *db/db* mice. They showed slight increases in blood glucose and body weight after NR supplementation. Adiponectin is a novel adipokine, which was expressed exclusively in adipose tissue. It has been reported that hypoadiponectinemia was associated with insulin resistance, and obesity may reduce plasma adiponectin levels (Maebuchi et al., 2003; Matsubara, 2004). We found that 400 mg/kg NR noticeably prevented the accumulation of subcutaneous fat and serum insulin, and significantly upregulated the serum adiponectin levels; however, NR was unable to significantly decrease blood glucose and body weight. This evidence supports the fact that NR might be effective for improving some DM-related symptoms.

Refractory wound healing in diabetes as a severe and chronic DM-related complication imposes a huge burden on patients. A large number of the population are victims of untimely and inefficient wound closure, especially patients with diabetes, owing to impaired angiogenesis (Azizi et al., 2019). Currently, there is no satisfactory treatment for diabetic wounds. Niacinamide, known as a form of NR, has been used as an annexing agent of skin-care products for many years. It seems to improve the nutritional status of skin cells (Carraway, 2004). A previous report suggested that niacinamide has the potential to remodel fibroblast matrix deposition and advance wound care, partly through the enhancement of cellular migration and proliferation (Wessels et al., 2014). Moreover, a clinical research found that a vitamin B complex including vitamin B_3_ showed positive effects on wound recovery after periodontal surgery (Neiva et al., 2005). Relevant to these findings, administration of NR did accelerate wound healing in *db/db* mice. NR also increased capillary densities which were indicated by CD31 staining at the wound site of *db/db* mice. However, further studies are needed to define the underlying mechanisms of how NR bolsters wound recovery and angiogenesis.

It is well known that EPCs participate in new blood vessel formation and tissue repair as precursors of endothelial cells (Zigdon-Giladi et al., 2015). Emerging data have indicated that recruitment of EPCs into ischemic tissues is one of the critical events in neovascularization (Weng et al., 2018). Wang et al. (2016) have reported that depletion of the NAD pool links diabetic patients to impairment of EPC mobilization. Supplementation of the NAD pool with nicotinamide contributes to increased numbers of circulating blood EPCs in diabetic patients (Wang et al., 2016). In addition, dietary vitamin intake such as vitamin B1 and vitamin D is dramatically associated with more circulating EPCs and better flow-mediated dilation (FMD) in T2DM patients than in controls (Wong et al., 2008; Gurses et al., 2014). Overall, enhancing the NAD pool may be a powerful factor for enhancing EPC activation under diabetic conditions. Notably, replenishing of NR, a vitamin B_3_ analog, has beneficial effects on EPC numbers in blood and BM with ischemia stroke models (Wang et al., 2011). Our present study indicates that the NAD concentration was downregulated in BM-EPCs of *db/db* mice. An upregulated NAD pool by NR treatment not only elevated the EPC numbers in circulating blood and BM but also enhanced EPC bioactivities, including capacity for tubule-like formation and adhesion. Thus, further research is required to understand the molecular mechanism underlying EPC improvements by NR in diabetic conditions.

Sirt1 as a NAD-dependent deacetylase plays a crucial role in mediating intracellular NAD levels for maintaining energy balance (Wang et al., 2014). PGC-1α is a transcriptional coactivator and has the only target of Sirt1 (Liao et al., 2019). A previous study favored the protective effect of Sirt1 and acetylated PGC-1α on the biological activity of EPCs (Wang et al., 2014). Our results demonstrate that a NR-replenished NAD pool was able to modulate Sirt1 and acetylated PGC-1α expressions in BM-EPCs of diabetic mice. As is well known, AMPK is recognized as a sensor of energy regulation to achieve glucose homeostasis and is widely expressed in all tissues (Liao et al., 2019). In a streptozotocin-induced mice model, AMPK activation was significantly decreased in BM-EPCs (Yu et al., 2016). Furthermore, an impaired EPC function is closely related to the inactivation of the AMPK pathway (Yu et al., 2016). It has been reported that Sirt1 upregulates the expression of phosphorylated AMPK under exposure to diabetic conditions (Karbasforooshan and Karimi, 2018). The Sirt1/AMPK pathway can participate in hepatic steatosis in obese mice (Liou et al., 2020). Our results indicate that treatment of the NAD substrate with NR increased the expression of phosphorylated AMPK in diabetic EPCs. More importantly, inhibition of Sirt1 signaling abolished the improvement of EPC function accompanied by upregulated phosphorylated AMPK by NR in BM-EPCs. Interestingly, AMPK inhibitors (compound C and C75) could partly reverse the beneficial effects of NR on EPC functions but had no effect on Sirt1 expression. Therefore, we confirmed that NR could enhance the EPC function by regulation of the Sirt1/AMPK pathway to achieve acceleration of wound healing in *db/db* mice.

VEGF is involved in the progression of wound repair and is recognized as important solely for mediating angiogenesis. Generally, downregulated VEGF levels in diabetic wound tissue are associated with impaired healing states, and enhancing pharmacologic VEGF may be a useful target (Galiano et al., 2004). Moreover, VEGF promotes EPC homing and repair of injured endothelial cells (Odent Grigorescu et al., 2017). We found that supplementing the NAD substrate with NR improved the disrupted expressions of VEGF in diabetic mice. However, inhibition of Sirt1 failed to reverse the enhanced expression of VEGF by NR treatment. In this regard, our results suggest that there is no direct association between increased VEGF concentration and the Sirt1/AMPK pathway in EPCs.

Wound healing is a sophisticated process responding to tissue damage, which is associated with numbers of interaction of various cell types, cytokines, growth factors, and other molecules (Jeffcoate and Harding, 2003; Shen et al., 2021). Therefore, improvement of only one aspect is not enough to dramatically promote wound healing. In this study, we only show that NR supplementation could regulate the EPC function to promote angiogenesis during wound healing. Future studies will be conducted to evaluate the NR function with different doses of NR.

In summary, we have shown evidence that increasing the NAD pool by NR accelerates wound healing and promotes angiogenesis in a diabetic mice model, which is possibly associated with improvement of the impaired EPC function *via* regulating the Sirt1/AMPK pathway. This report implies new applications for NR supplementation in the daily diet of diabetic individuals to achieve early prevention of diabetic complications. Further research aimed at defining the mechanisms of NR on diabetic complications is required.

## Data Availability

The raw data supporting the conclusion of this article will be made available by the authors, without undue reservation.
