# Supplementary material for: Nicotinamide Riboside Enhances Endothelial Precursor Cell Function to Promote Refractory Wound Healing Through Mediating the Sirt1/AMPK Pathway
Source: Front Pharmacol. 2021 May 12;12:671563. doi: 10.3389/fphar.2021.671563 (PMC8149616; doi:10.3389/fphar.2021.671563)
Supplement: Supplementary file 1 [file DataSheet1.docx]

**Supplementary material**

Nicotinamide Riboside Enhances Endothelial Precursor Cell Function to Promote Refractory Wound Healing Through Mediating the Sirt1/AMPK Pathway

**Supplementary Figure 1**


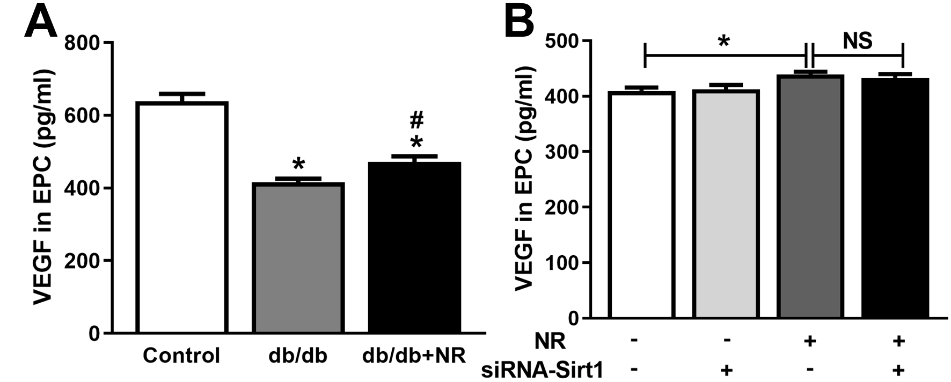


**VEGF concentration in EPCs.** (A) The level of VEGF in EPCs was detected by ELISA. ^*^*P* <0.05 *vs.* Control; ^#^*P* <0.05 *vs.* *db/db*. (B) *Db/db* mice received the NR supplied food for 12 weeks, then BM-EPCs were isolated and transfected with siRNA targeting Sirt1. The concentration of VEGF in EPCs was measured. ^*^*P* <0.05. Values are mean ± SEM, (n=5). NS, no significance.

**Supplementary Figure 2**


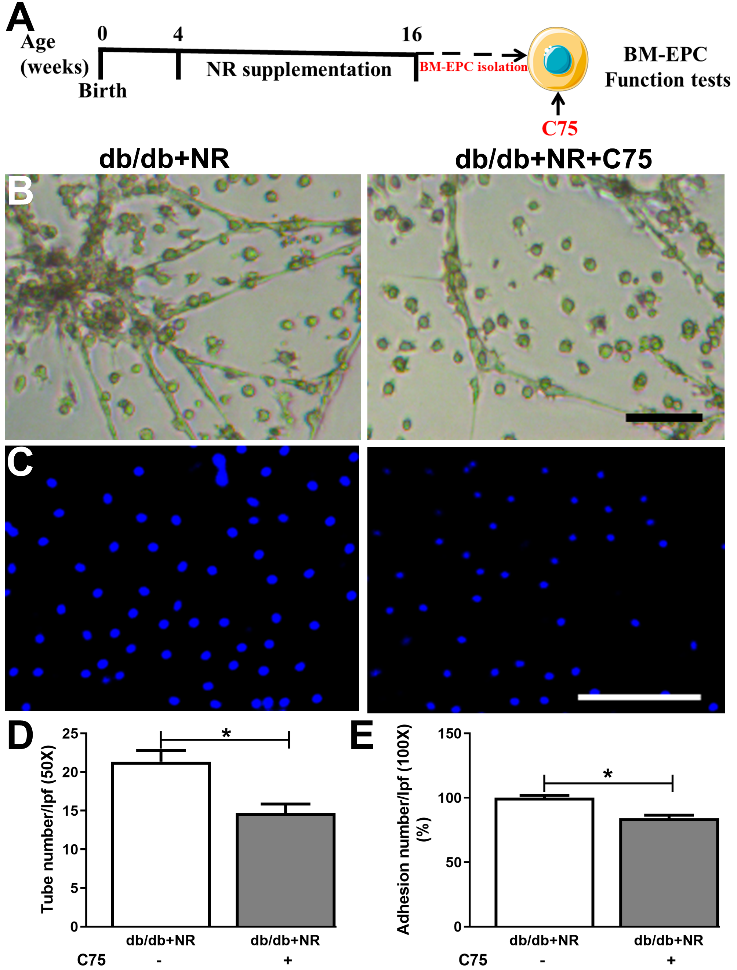


**Inhibition of AMPK with C75 abolished the protective effects of NR supplement on BM-EPC function in *db/db* mice.** (A) Illustration of the experimental protocol. *Db/db* mice were treated with NR for 12 weeks, then BM-EPCs were isolated and stimulated with C75 (40 μg/ml). Tube formation (B) and adhesion (C) assay in BM-EPCs treated with NR in the presence or absence of C75. Quantitated analysis of tube number (D) and adhesion number (E). B: 50×; scale bar, 100μm; C: 100×; scale bar, 100μm. ^*^*P* <0.05. Values are mean ± SEM, (n=3).
